# Emotions and Steroid Secretion in Aging Men: A Multi—Study Report

**DOI:** 10.3389/fpsyg.2017.01722

**Published:** 2017-09-29

**Authors:** Andreas Walther, Patricia Waldvogel, Emilou Noser, Jessica Ruppen, Ulrike Ehlert

**Affiliations:** ^1^Clinical Psychology and Psychotherapy, University of Zurich, Zurich, Switzerland; ^2^University Research Priority Program (URPP) Dynamics of Healthy Aging, University of Zurich, Zurich, Switzerland; ^3^Biological Psychology, Technische Universität Dresden, Dresden, Germany; ^4^Central European Network on Fatherhood (CENOF), Headquarters at the University of Vienna, Vienna, Austria

**Keywords:** aging, emotional experience, steroid secretion, testosterone, cortisol, anxiety, depression, vital exhaustion

## Abstract

Although aging increases the risk of cognitive and socioemotional deterioration, it has also been shown to be accompanied by an increase in experienced positive emotions and a decrease in negative emotions. Steroid hormones and age-related alterations in secretion patterns have been suggested to play a crucial role in these age-related changes in emotional experience. Importantly, previous studies identified effects of neuroactive hormones on age-related alterations in emotional experience, which vary by sex and depression levels. Therefore, in three independent cross-sectional studies including a total of 776 men, we examined age-related differences in emotional experience and subsequently the moderation effect of steroid hormones. Sample one consisted of 271 self-reporting healthy (SRH) men aged between 40 and 75 years, while sample two comprised 121 men in the identical age range but only including vitally exhausted (VE) men. Sample three included 384 men aged between 25 and 78 years who reported having fathered (FA) at least one child. For the SRH men, age was negatively associated with anxiety symptoms and aggression, while negative trends emerged for depressive symptoms. In VE men, age was negatively associated with depressive symptoms and positively associated with aggression and positive emotions. For FA men, anxiety symptoms and aggression were negatively associated with age. Age trends of steroid hormones and identified moderation effects are reported. However, with adjustment for multiple comparisons, most of the significant associations fade and the reported associations need to be regarded as exploratory starting points for the further investigation of age-related alterations in emotional experience and their relation to steroid secretion. Overall, the results indicate that salivary cortisol might be a moderator of the association between age and symptoms of anxiety for SRH and VE men, while salivary testosterone seems to moderate the association between age and symptoms of anxiety or depression in VE and FA men, respectively. Both hair cortisol and progesterone seem to influence age-related alterations in anger experience. Age-related alterations in the hypothalamic-pituitary-adrenal (HPA) axis and the hypothalamic-pituitary-gonadal (HPG) axis emerge as promising avenues to further investigate the decrease in experienced negative emotions in aging men.

## Introduction

Aging has been related to cognitive and socioemotional decline in several longitudinal studies (Carlo et al., 2000; Park and Bischof, 2013). Various cognitive and socioemotional functions undergo age-related changes, and although most functions deteriorate continuously with age (Ruffman et al., 2008), some show improvement. Emotional experience seems to be relatively unaffected by age or even appears to show an increase in terms of the total amount of experienced positive emotions (Carstensen et al., 2011) or general life satisfaction (Gana et al., 2013). With age, an increase in positive feelings such as, happiness, calmness, relaxedness, and enthusiasm, and simultaneously a significant decrease in negative feelings such as, boredom, fatigue, and a decreasing trend for anger have been reported (English et al., 2014). This age-related positivity effect in emotional experience favoring positive over negative stimuli has been well-documented in the past 15 years (Reed and Carstensen, 2012; Mammarella et al., 2016, 2017; Mather, 2016), and has important health implications since survival rates were shown to be related to positive affectivity in older individuals (Steptoe and Wardle, 2011). In line with these findings, Baer et al. reported a continuous decrease in the prevalence of depression with increasing age (Baer et al., 2013), while a progressive age-related downward trend in worry frequency in individuals with and without general anxiety disorders was demonstrated (Miloyan and Pachana, 2015). Wise reasoning is another psychological construct related to this positivity effect and it seems to increase with more life experiences (Grossmann et al., 2013). Furthermore, older individuals show preferences for positive low-arousal affect (calm, peaceful, relaxed) over positive high-arousal affect (excited, proud) matching with a state of wise reasoning (Scheibe et al., 2013). As other potential links between age and lower negative affect, increased acceptance of negative emotional experience or selective narrowing of social networks have been identified (Shallcross et al., 2013; English and Carstensen, 2014).

Neurobiological models indicate important biological underpinnings of age-related changes in emotion perception (Ruffman et al., 2008; Ebner and Fischer, 2014). Steroid hormones have been suggested to moderate the association between age and socioemotional functions (Ebner et al., 2014). A possible link between age-related hormonal changes and changes in emotional experience in aging individuals adds relevance to the examination of the moderating potential of the endocrine system in this research area (Walther and Ehlert, 2015; Schiller et al., 2016). In men over the age of 40 years, sex steroids (e.g., testosterone [T], dehydroepiandrosterone [DHEA], estradiol [E2]) continuously decline (Feldman et al., 2002; Frost et al., 2013; Walther et al., 2016), while cortisol (C) has been shown to increase with age (Karlamangla et al., 2013; Nater et al., 2013; Miller et al., 2016). However, T, as the end product of the hypothalamic-pituitary-gonadal (HPG) axis, has been positively related to aggression, dominance, and anger in men, while inverse associations have been shown for depressive and anxiety symptoms (Edinger and Frye, 2005; Wirth and Schultheiss, 2007; Carrier et al., 2015; Walther et al., 2017c). In contrast, higher levels of C, the primary effector of the biological stress response released via the hypothalamic-pituitary-adrenal (HPA) axis, have been associated with more depressive and anxious symptoms as well as higher scores for worrying (Ehlert et al., 2001; Takahashi et al., 2005; Mantella et al., 2008), although there is substantial conflicting literature indicating differences between symptoms of anxiety, anxiety disorders, and age as well as sex of examined subjects (Steudte et al., 2011; Yehuda and Seckl, 2011; Hek et al., 2013; Steudte-Schmiedgen et al., 2017). Therefore, an examination of potential associations between age-related changes in emotional experience and steroid secretion might yield crucial insights into the underlying neurobiology of this shift toward a more positive emotional experience with increasing age.

To examine the potential moderation effects of steroid secretion and age-related changes in emotional experience, we investigated three independent male samples with regard to steroid secretion and emotional experience. The first sample consisted of 271 self-reporting healthy (SRH) men between the ages of 40 and 75 from the Men's Health 40+ study (Walther et al., 2017a), while the second sample encompassed 121 men in the identical age range from the Men Stress 40+ study with a minimum score of 4 on the Maastricht Vital Exhaustion Questionnaire (MVEQ, Appels et al., 1987). Vital exhaustion (VE) is defined as a psychological state characterized by excessive fatigue, loss of vigor, increased irritability, and feelings of demoralization, while depressed mood, low self-esteem, or feelings of guilt, which are core symptoms of depression, are not predictive for VE (van Diest and Appels, 1991; Appels, 2004). Furthermore, VE has been shown to be associated with neuroticism, social anxiety, and hostility (van Zijderveld et al., 2013). In patients with chronic heart failure fatigue, cognitive-affective depressive symptoms, sleep difficulties, and a lack of concentration were predominantly observed (Smith et al., 2009). Finally, as previous studies revealed a significant influence of fatherhood on sex steroid secretion (Gettler et al., 2011; Perini et al., 2012), the third sample comprised 384 men aged between 25 and 78 years from a large-scale fatherhood study, only including men who reported having fathered at least one biological or non-biological child (Waldvogel and Ehlert, 2016). The comparison of these three samples is of major interest as sample 1 (SRH men) and sample 2 (VE men) compare men of the identical age range (40 to 75 years), very similar levels of socioeconomic status and environmental conditions and only differing by their perceived health status as either very healthy or vital exhausted. Exhaustion has been related to alterations in steroid secretion (Kudielka et al., 2006; Bellingrath et al., 2008; Snyder et al., 2016) and emotion experience (Koertge et al., 2007; Doyle et al., 2011) and therefore different associations between steroid hormones and the age-related alterations in emotion experience are expected in VE men compared to SRH men. Furthermore, decreased androgen secretion has been reported in men after becoming a father and was associated with a decreased relationship quality due to missing tenderness (Gettler et al., 2011; Perini et al., 2012). These findings indicate that changes in circulating levels of androgens may be associated with “tend and befriend” behavior, what might contribute to better emotional experience with age (Van Anders, 2013). In the following, we describe the methods and findings of these three studies on the hormonal contribution to age-related changes in emotional perception. We conclude the report by integrating the findings into a comprehensive framework on the role of steroid secretion in age-related changes in emotional experience in men.

## Study 1: men's health 40+

### Participants and procedure

A total of 271 men between the ages of 40 and 75 years participated in the Men's Health 40+ study in the year 2014. They were recruited from the German-speaking part of Switzerland via flyers and web pages. The study only included SRH men, which was controlled for by the first question of the Short Form 36 (SF-36) Health Survey: “How would you describe your current health status?” (Brazier et al., 1992). 35.8% of the participants indicated the response option “very good,” while 54.6 and 9.6% responded with “good” and “fair,” respectively. None of the participants indicated the response options “bad” or “very bad.” Participants had a mean age of 57.1 (*SD* = 10.7) years and a mean body mass index (BMI) of 25.4 (*SD* = 3.4). Only 8.2% of participants reported being regular smokers. In general, a relatively high socioeconomic status was observed, as reflected by the mean education level (88.1% higher secondary school or higher) and income level (48.3% earning more than CHF100,000/year what corresponds to 104'134 USD).

Participants independently completed psychometric questionnaires online. After completing the psychometric test battery, they were invited to a biological examination, where saliva and hair samples were obtained using standardized procedures. All participants provided written informed consent, and the study protocol was approved by the local Ethics Committee of the Faculty of Arts at the University of Zurich. Prior published data of this sample can be reviewed here (Walther et al., 2016, 2017a,b; Noser et al., 2017).

### Psychometric measures

The German version of the Brief Symptom Inventory—18 (BSI-18) was used to measure self-reported psychological distress focusing on symptoms of anxiety, depression, and somatization (Zabora et al., 2001; Franke et al., 2011). The questionnaire consists of 18 items distributed over the three subscales depression, anxiety, and somatization. Each subscale comprises six items rated on a 5-point Likert scale from 0 (not at all) to 4 (always) and participants were asked about symptoms experienced in the last 7 days. To obtain indicators of negative emotions, only the scales depression and anxiety were analyzed.

Aggression was measured via the German version of the Buss Perry Aggression Questionnaire (BPAQ) (Buss and Perry, 1992; Herzberg, 2003). The questionnaire comprises 29 items rated on a 5-point Likert scale from 1 (extremely uncharacteristic of me) to 5 (extremely characteristic of me) distributed over the four scales physical aggression, verbal aggression, anger, and hostility. All four scales were analyzed, as although only anger is a negative emotion in terms of subjective experience and social evaluation (Averill, 1982), aggressive behavior such as, physical or verbal aggression or hostile behavior can be considered a proxy for anger given that anger drives many, if not most, forms of aggression (Averill, 1982).

### Endocrine measures

Standardized saliva sampling took place starting at 8:00 am at the laboratory of the Psychological Institute of the University of Zurich. Participants were asked to fast overnight and to refrain from exercise or engaging in sexual activity for 24 h before testing. Moreover, they were requested not to brush their teeth or smoke and to only drink water on the morning before providing the saliva sample. Saliva was collected via a commercially available sampling device employing the passive drool method (SaliCaps, IBL International GmbH, Hamburg, Germany). Saliva samples were obtained during a 15-min time period and were stored at –20°C until required for biochemical analysis at the Biochemical Laboratory of the Institute of Psychology at the University of Zurich. All steroid hormones including salivary C (sC), salivary T (sT), salivary DHEA (sDHEA), salivary E2 (sE2), and salivary progesterone (sP) were quantified with standard enzyme immunoassays (retrievable under: http://www.ibl-international.com/ende/; Lewis, 2006; Chiappin et al., 2007). Intra- and inter-assay coefficients for the analyzed steroid hormones were below 10%, while sensitivity was 0.003 μg/dL for sC, 0.083 pg/ml for sT, or 0.6 pg/ml for sE2 according to Immuno-Biological Laboratories, Inc, IBL-America.

In addition, hair samples of T (hT), DHEA (hDHEA), P (hP), C (hCrtsl), and cortisone (hCrtson) were obtained from 211 participants to determine the concentrations of steroid hormones in hair. Therefore, 2 cm segments of hair from the posterior vertex proximal to the scalp were obtained, to reflect the steroid secretion of the last 2 months (assuming an average hair growth of 1 cm per month). For biochemical analysis with liquid chromatography-mass spectrometry, samples were sent to the Dresden Lab (Stalder and Kirschbaum, 2012; Gao et al., 2013, 2016; Stalder et al., 2017). Intra- and inter-assay coefficients for the analyzed steroid hormones were below 10%, while sensitivity was 0.5 pg/mg for C, 0.15 pg/mg for T, or 0.05 pg/mg for DHEA.

### Covariates

Covariates included in the analyses were BMI, alcohol, or tobacco consumption, medication intake, general health status and health effort, recent physical activity, recent sexual activity, starting time of the examination, waking time of the participant, gum bleeding during the last days, and elevated stress during the last week. For hair steroids, the following covariates were additionally controlled for: month of hair sampling, times of hair washing, hair color, artificial hair coloring, hair spray (excluding: waking time of the participant, elevated stress during the last week). We controlled for these covariates as they were previously reported to significantly influence steroid measurements in saliva or hair (Walther et al., 2016; Stalder et al., 2017).

### Statistical analysis

Pearson's zero-order correlations (*r*) were performed to examine the association between age and parameters for negative emotions and steroid hormones. Subsequently, partial correlation analyses were conducted including the introduced covariates. Next, separate moderation analyses were conducted for the association between age and negative emotions moderated by steroid hormones using PROCESS for SPSS (Hayes, 2013). Additionally, the potential covariates were included for the moderation analyses. All analyses were carried out using SPSS, version 23.0 (IBM SPSS Statistics, Armonk, NY, USA). The level of significance was set at α = 0.05.

### Results

Sample characteristics and descriptive statistics of salivary and hair analytes are summarized in Table 1. Pearson's correlation analyses showed significant associations between age (ranging from 40 to 75 years) and different psychometric indicators of negative emotions. The BSI-18 subscale anxiety was negatively associated with age (*r* = −0.188, *p* = 0.001), while for depression, only a negative trend emerged (*r* = −0.098, *p* = 0.053). With regard to the aggression questionnaire, age was significantly negatively associated with anger (*r* = −0.166, *p* = 0.003) and physical aggression (*r* = −0.133, *p* = 0.014), and positively associated with verbal aggression (*r* = 0.113, *p* = 0.032), but was not associated with hostility (*r* = −0.019, *p* = 0.381). Age was negatively associated with sT (*r* = −0.345, *p* < 0.001), sDHEA (*r* = −0.385, *p* < 0.001), sE2 (*r* = −0.208, *p* < 0.001) and sP (*r* = −0.276, *p* < 0.001) and positively associated with sC (*r* = 0.145, *p* = 0.009). Employing partial correlation analyses including the introduced set of covariates did not change associations with age (anxiety: *r*_*p*_ = −0.191, *p* = 0.001; depression: *r*_*p*_ = −0.087, *p* = 0.079; anger: *r*_*p*_ = −0.108, *p* = 0.042; physical aggression: *r*_*p*_ = −0.116, *p* = 0.031; verbal aggression: *r*_*p*_ = 0.127, *p* = 0.021; sT: *r*_*p*_ = −0.236, *p* < 0.001; sDHEA: *r*_*p*_ = −0.301, *p* < 0.001; sE2: *r*_*p*_ = −0.191, *p* = 0.001; sP: *r*_*p*_ = −0.203, *p* = 0.001; sC: *r*_*p*_ = 0.128, *p* = 0.024; see Table 2). However, for the hair steroids, a significant association with age only emerged for hT (hT: *r* = −0.320, *p* < 0.001), while all other hair steroids failed to show age-related associations (hCrtsl: *r* = 0.038, *p* = 0.291; hCrtson: *r* = −0.016, *p* = 411; hDHEA: *r* = 0.030, *p* = 0.334; hP: *r* = −0.057, *p* = 0.204). The pattern changed when including covariates into the correlation analyses, with partial correlation analyses revealing the following age-related associations with hair steroids: hCrtsl: *r*_*p*_ = 0.139, *p* = 0.031; hCrtson: *r*_*p*_ = 0.100, *p* = 0.090; hT: *r*_*p*_ = −0.187, *p* = 0.006; hP: *r*_*p*_ = −0.118, *p* = 0.058; hDHEA: *r*_*p*_ = −0.026, *p* = 0.366.

**Table 1:** Means and standard deviations or percentage frequencies of demographics, psychometric, and endocrine measures in the different studies.

	**Study 1: Men's health 40+**	**Study 2: Men stress 40+**	**Study 3: Fatherhood**
	*N* = 271	*N* = 121	*N* = 384
**DEMOGRAPHICS**			
Age	57.51 (10.74)	52.69 (8.35)	43.75 (10.72)
BMI	25.37 (3.06)	25.79 (3.86)	25.28 (3.57)
Smoking (%)	8.2	11.6	12.0
Alcohol (%)	79.6	80.2	85.9
Education (%)	88.1 ≥ higher secondary school	62.0 ≥ university or applied sciences degree	59.1 ≥ tertiary education
Income	48.3% > CHF 100,000/year	50.0% > CHF 117,000/year	CHF 7,565/month (an estimate of CHF 90,779/year)
**BRIEF SYMPTOM INVENTORY (BSI)**			
BSI—Anxiety	2.37 (2.67)	4.43 (3.02)	2.50 (2.59)
BSI—Depression	1.63 (2.69)	4.02 (3.59)	2.21 (3.07)
BSI—Aggression	–	–	2.12 (2.31)
**BUSS PERRY AGGRESSION QUESTIONNAIRE (BPAQ)**			
BPAQ—Physical	2.28 (0.41)	2.28 (0.39)	–
BPAQ—Verbal	2.37 (0.57)	2.56 (0.64)	–
BPAQ – Anger	2.21 (0.43)	2.11 (0.38)	–
BPAQ – Hostility	2.26 (0.46)	2.35 (0.46)	–
**POSITIVE AND NEGATIVE AFFECT SCHEDULE (PANAS)**			
PANAS—PA	–	31.44 (6.47)	–
PANAS—NA	–	21.55 (6.25)	–
**SALIVARY ANALYTES**			
sT (pg/ml)	67.37 (26.72)	65.80 (26.95)	56.20 (25.99)
sDHEA (pg/ml)	256.25 (224.26)	148.63 (154.27)	–
sE2 (pg/ml)	1.32 (0.999)	1.66 (0.91)	1.52 (1.46)
sP (pg/ml)	28.43 (18.79)	39.20 (42.81)	–
sC (nmol/L)	18.27 (8.16)	7.87 (4.17)	17.05 (8.95)
**HAIR ANALYTES**			
hT (pg/mg)	0.90 (0.76)	5.01 (6.98)	–
hDHEA (pg/mg)	1.07 (0.92)	16.89 (12.71)	–
hP (pg/mg)	1.86 (1.22)	2.07 (2.39)	–
hCrtson (pg/mg)	24.57 (16.39)	31.58 (28.58)	–
hCrtsl (pg/mg)	7.96 (6.32)	21.94 (29.84)	–

*Table entries are means and, in parentheses, standard deviations, or percentage frequencies where noted. Absolut numbers vary slightly among different variables. T, Testosterone; C, Cortisol; DHEA, Dehydroepiandrosterone; P, Progesterone; E2, Estradiol; sC, salivary C; sT, salivary T; sDHEA, salivary DHEA; sP, salivary P; sE2, salivary E2; hT, hair T; hCrtsl, hair C; hDHEA, hair DHEA; hair P, hP; hair cortisone, hCrtson; BSI, Brief Symptom Inventory; PANAS, Positive and Negative Affect Schedule; BPAQ, Buss-Perry Aggression Questionnaire*.

**Table 2:** Partial correlations for age and emotion experience and steroid hormones.

	**Study 1: Men's health 40+**	**Study 2: Men stress 40+**	**Study 3: Fatherhood**
**AGE**			
Anxiety (BSI)	**−0.191**	0.110	**−0.113**
	**0.001**	0.131	**0.030**
Depression (BSI)	−0.087	−0.117	−0.011
	0.079^t^	0.116	0.831
Aggression (BSI)	–	–	**−0.175**
			**0.001**
Physical aggression (BPAQ)	**−0.116**	−0.160	–
	**0.031**	0.051^t^	
Verbal aggression (BPAQ)	**0.127**	**0.180**	–
	**0.021**	**0.033**	
Anger (BPAQ)	**−0.108**	0.131	–
	**0.042**	0.090^t^	
Hostility (BPAQ)	0.007	0.012	–
	0.458	0.453	
Positive affect (PANAS)	–	0.149	–
		0.064^t^	
Negative affect (PANAS)	–	0.001	–
		0.495	
sT	**−0.236**	**−0.246**	**−0.176**
	**0.000**	**0.006**	**0.001**
sDHEA	**−0.301**	**−0.338**	–
	**0.000**	**0.000**	
sE2	**−0.191**	0.008	**0.151**
	**0.001**	0.468	**0.004**
sP	**−0.203**	**−0.193**	–
	**0.001**	**0.024**	
sC	**0.128**	−0.125	−0.040
	**0.024**	0.101	0.441
hT	**−0.187**	**−0.283**	–
	**0.006**	**0.004**	
hDHEA	0.026	−0.133	–
	0.366	0.110	
hP	−0.118	−0.032	–
	0.058^t^	0.386	
hCrtson	0.100	−0.109	–
	0.090^t^	0.127	
hCrtsl	**0.139**	0.041	–
	**0.031**	0.336	

*Upper values in the compartments represent correlation coefficients and values below represent level of significance. Bold correlations are significant at p < 0.05. T, Testosterone; C, Cortisol; DHEA, Dehydroepiandrosterone; P, Progesterone; E2, Estradiol; sC, salivary C; sT, salivary T; sDHEA, salivary DHEA; sP, salivary P; sE2, salivary E2; hT, hair T; hCrtsl, hair C; hDHEA, hair DHEA; hair P, hP; hair cortisone, hCrtson; BSI, Brief Symptom Inventory; PANAS, Positive and Negative Affect Schedule; BPAQ, Buss-Perry Aggression Questionnaire*.

Moderation analyses for the association between age and parameters of negative emotional state including covariates are summarized in Table 3. The salivary analytes tested as moderators were sC, sT, sDHEA, sE2, and sP. A significant moderation effect of sC on the association between age and the BSI-18 anxiety scale emerged (*R*^2^ = 0.4146, *p* < 0.001; B = −0.0036, SE = 0.0018, *p* = 0.0453, *R*^2^ change = 0.0139). No other significant moderation effect emerged for the association between age and anxiety or depression for salivary analytes as moderators (see Table 3). For the association between age and aggression (physical and verbal), trends for moderation effects were observed for sP (Physical aggression: *R*^2^ = 0.1013, *p* = 0.084, B = 0.0002, SE = 0001, *p* = 0.0685, *R*^2^ change = 0.0124; Verbal aggression: *R*^2^ = 0.0687, *p* = 0.1140; B = 0.0003, SE = 0.0002, *p* = 0.0896, *R*^2^ change = 0.0139). No other moderation effects were observed (see Table 3).

**Table 3:** Moderation analyses for steroid hormones on the association between age and emotion experience.

	**Study 1: Men's health 40**+				**Study 2: Men stress 40**+				**Study 3: Fatherhood**			
	***R*^2^**	**p-model**	**B**	**p-int**	***R*^2^**	**p-model**	**B**	**p-int**	***R*^2^**	**p-model**	**B**	**p-int**
**sT**												
Age:BSI-Anxiety	0.1472	0.0016	−0.0005	0.3678	0.4022	0.0001	−0.0027	0.0225^*^	0.1165	0.0000	0.0299	0.0511^t^
Age:BSI-Depression	0.1081	0.0451	−0.0002	0.7650	0.4070	0.0001	−0.0023	0.1014	0.1461	0.0000	0.0412	0.0210^*^
Age:BSI-Aggression	x	x	x	x	x	x	x	x	0.1319	0.0000	0.0025	0.8537
Age:BPAQ-Physical	0.0824	0.2390	0.0000	0.6071	0.2415	0.0950	−0.0013	0.4036	x	x	x	x
Age:BPAQ -Verbal	0.0874	0.1819	−0.0001	0.6327	0.2310	0.1292	0.0004	0.7787	x	x	x	x
Age:BPAQ -Anger	0.1111	0.0384	0.0001	0.4731	0.3362	0.0024	−0.0015	0.5085	x	x	x	x
Age:BPAQ-Hostility	0.1123	0.0352	0.0001	0.5675	0.3134	0.0068	0.0010	0.6443	x	x	x	x
Age:PANAS-PA	x	x	x	x	0.3038	0.0102	0.0029	0.2817	x	x	x	x
Age:PANAS-NA	x	x	x	x	0.3762	0.0003	0.0001	0.9798	x	x	x	x
**sDHEA**												
Age:BSI-Anxiety	0.1512	0.0013	0.0000	0.5724	0.3998	0.0000	−0.0002	0.4071	x	x	x	x
Age:BSI-Depression	0.1107	0.0395	−0.0001	0.2686	0.3689	0.0003	−0.0001	0.7650	x	x	x	x
Age:BSI-Aggression	x	x	x	x	x	x	x	x	x	x	x	x
Age:BPAQ-Physical	0.0788	0.2954	0.0000	0.4515	0.3122	0.0046	0.0001	0.8676	x	x	x	x
Age:BPAQ -Verbal	0.1031	0.0715	0.0000	0.4544	0.2237	0.1280	0.0003	0.2902	x	x	x	x
Age:BPAQ -Anger	0.1123	0.0374	0.0000	0.4374	0.3318	0.0018	−0.0001	0.8520	x	x	x	x
Age:BPAQ -Hostility	0.0977	0.1022	0.0000	0.6535	0.2987	0.0084	0.0005	0.2392	x	x	x	x
Age:PANAS-PA	x	x	x	x	0.2924	0.0110	0.0002	0.6601	x	x	x	x
Age:PANAS-NA	x	x	x	x	0.3719	0.0002	−0.0001	0.8460	x	x	x	x
**sE2**												
Age:BSI-Anxiety	0.1494	0.0012	−0.0006	0.9681	0.3830	0.0001	−0.0408	0.2942	0.1024	0.0001	−0.0061	0.6223
Age:BSI-Depression	0.1176	0.0204	−0.0020	0.9045	0.3775	0.0002	−0.0348	0.4528	0.1179	0.0000	0.0012	0.9328
Age:BSI-Aggression	x	x	x	x	x	x	x	x	0.1268	0.0000	−0.0102	0.3485
Age:BPAQ -Physical	0.0808	0.2527	0.0005	0.8382	0.3605	0.0004	0.0860	0.0841^t^	x	x	x	x
Age:BPAQ -Verbal	0.0845	0.2073	−0.0004	0.9136	0.2302	0.1054	0.0573	0.2342	x	x	x	x
Age:BPAQ -Anger	0.1064	0.0512	0.0000	0.9867	0.3414	0.0011	0.0675	0.3471	x	x	x	x
Age:BPAQ -Hostility	0.1001	0.0794	−0.0019	0.5024	0.3418	0.0011	0.1168	0.0922^t^	x	x	x	x
Age:PANAS-PA	x	x	x	x	0.2922	0.0111	−0.0046	0.9589	x	x	x	x
Age:PANAS-NA	x	x	x	x	0.3710	0.0002	0.0427	0.5984	x	x	x	x
**sP**												
Age:BSI-Anxiety	0.1531	0.0012	−0.0009	0.2409	0.3841	0.0001	−0.0008	0.5020	x	x	x	x
Age:BSI-Depression	0.1002	0.0871	−0.0008	0.3010	0.3662	0.0003	−0.0001	0.9650	x	x	x	x
Age:BSI-Aggression	x	x	x	x	x	x	x	x	x	x	x	x
Age:BPAQ -Physical	0.1013	0.0848	0.0002	0.0685^t^	0.2836	0.0157	0.0009	0.5872	x	x	x	x
Age:BPAQ -Verbal	0.0967	0.1140	0.0003	0.0896^t^	0.2159	0.1598	0.0002	0.8790	x	x	x	x
Age:BPAQ -Anger	0.1166	0.0289	0.0001	0.5498	0.3415	0.0011	−0.0012	0.6108	x	x	x	x
Age:BPAQ -Hostility	0.1083	0.0530	0.0000	0.8764	0.2956	0.0097	0.0015	0.4960	x	x	x	x
Age:PANAS-PA	x	x	x	x	0.2943	0.0102	0.0000	0.9959	x	x	x	x
Age:PANAS-NA	x	x	x	x	0.3833	0.0001	−0.0039	0.1331	x	x	x	x
**sC**												
Age:BSI-Anxiety	0.1719	0.0002	−0.0036	0.0453^*^	0.4062	0.0000	−0.0152	0.0262^*^	0.1019	0.0001	−0.0038	0.8224
Age:BSI-Depression	0.0985	0.1017	−0.0015	0.4010	0.3701	0.0002	−0.0024	0.7761	0.1162	0.0000	0.0048	0.8103
Age:BSI-Aggression	x	x	x	x	x	x	x	x	0.1248	0.0000	−0.0057	0.6997
Age:BPAQ -Physical	0.0808	0.2854	0.0002	0.4820	0.3534	0.0006	−0.0130	0.1421	x	x	x	x
Age: BPAQ -Verbal	0.0886	0.1906	0.0007	0.0860^t^	0.2137	0.1698	−0.0005	0.9574	x	x	x	x
Age:BPAQ -Anger	0.1226	0.0143	−0.0003	0.3036	0.3446	0.0010	−0.0172	0.1762	x	x	x	x
Age:BPAQ -Hostility	0.1046	0.0720	0.0003	0.3866	0.3099	0.0051	0.0050	0.6912	x	x	x	x
Age:PANAS-PA	x	x	x	x	0.2888	0.0128	−0.0033	0.8346	x	x	x	x
Age:PANAS-NA	x	x	x	x	0.4051	0.0000	−0.0242	0.0859^t^	x	x	x	x
**hT**												
Age:BSI-Anxiety	0.1531	0.0145	0.0201	0.3482	0.3864	0.0001	0.0100	0.1873	x	x	x	x
Age:BSI-Depression	0.0943	0.3196	0.0089	0.6753	0.4157	0.0000	0.0059	0.4885	x	x	x	x
Age:BSI-Aggression	x	x	x	x	x	x	x	x	x	x	x	x
Age:BPAQ -Physical	0.1404	0.0337	0.0033	0.3176	0.2607	0.0167	0.0105	0.2772	x	x	x	x
Age:BPAQ-Verbal	0.0972	0.2917	−0.0005	0.9079	0.1250	0.5668	−0.0014	0.8852	x	x	x	x
Age:BPAQ-Anger	0.1833	0.0018	0.0028	0.3843	0.3372	0.0007	0.0047	0.7437	x	x	x	x
Age:BPAQ-Hostility	0.1376	0.0397	0.0018	0.6300	0.2867	0.0062	0.0067	0.6406	x	x	x	x
Age:PANAS-PA	x	x	x	x	0.2605	0.0168	0.0209	0.2536	x	x	x	x
Age:PANAS-NA	x	x	x	x	0.3719	0.0001	0.0394	0.0168^*^	x	x	x	x
**hDHEA**												
Age:BSI-Anxiety	0.1333	0.0423	−0.0060	0.7751	0.3694	0.0003	−0.0032	0.3359	x	x	x	x
Age:BSI-Depression	0.1028	0.2085	−0.0088	0.6658	0.3934	0.0001	0.0009	0.8064	x	x	x	x
Age:BSI-Aggression	x	x	x	x	x	x	x	x	x	x	x	x
Age:BPAQ-Physical	0.1398	0.0296	0.0033	0.2984	0.3030	0.0050	0.0009	0.8242	x	x	x	x
Age:BPAQ-Verbal	0.1104	0.1505	−0.0008	0.8498	0.1290	0.6066	−0.0014	0.7512	x	x	x	x
Age:BPAQ-Anger	0.2003	0.0004	−0.0024	0.4477	0.3776	0.0002	0.0119	0.0526^t^	x	x	x	x
Age:BPAQ-Hostility	0.1642	0.0058	−0.0009	0.8088	0.2989	0.0059	−0.0047	0.4513	x	x	x	x
Age:PANAS-PA	x	x	x	x	0.2830	0.0109	−0.0054	0.4867	x	x	x	x
Age:PANAS-NA	x	x	x	x	0.3444	0.0008	0.0061	0.3993	x	x	x	x
**hP**												
Age:BSI-Anxiety	0.1316	0.0435	0.0026	0.8499	0.3633	0.0003	0.0007	0.9762	x	x	x	x
Age:BSI-Depression	0.1012	0.2145	0.0199	0.1316	0.4058	0.0000	0.0187	0.4486	x	x	x	x
Age:BSI-Aggression	x	x	x	x	x	x	x	x	x	x	x	x
Age:BPAQ-Physical	0.1502	0.0139	0.0044	0.0315^*^	0.2895	0.0086	−0.0100	0.7236	x	x	x	x
Age:BPAQ-Verbal	0.1449	0.0197	0.0082	0.0044^**^	0.1263	0.6284	−0.0034	0.9045	x	x	x	x
Age:BPAQ-Anger	0.2147	0.0001	0.0023	0.2521	0.3509	0.0006	−0.0178	0.6646	x	x	x	x
Age:BPAQ-Hostility	0.1366	0.0334	0.0015	0.5164	0.3053	0.0046	−0.0294	0.4728	x	x	x	x
Age:PANAS-PA	x	x	x	x	0.2571	0.0276	−0.0023	0.6722	x	x	x	x
Age:PANAS-NA	x	x	x	x	0.3366	0.0012	−0.0136	0.7772	x	x	x	x
**hCrtsn**												
Age:BSI-Anxiety	0.1412	0.0239	−0.0008	0.4235	0.3307	0.0000	0.0010	0.4696	x	x	x	x
Age:BSI-Depression	0.1066	0.1676	−0.0011	0.2718	0.3304	0.0000	0.0004	0.7142	x	x	x	x
Age:BSI-Aggression	x	x	x	x	x	x	x	x	x	x	x	x
Age:BPAQ-Physical	0.1268	0.0598	0.0001	0.5739	0.3041	0.0001	−0.0008	6077	x	x	x	x
Age:BPAQ-Verbal	0.1060	0.1764	0.0000	0.9583	0.1974	0.0270	−0.0026	0.0817^t^	x	x	x	x
Age:BPAQ-Anger	0.2078	0.0002	0.0003	0.0331^*^	0.3385	0.0000	−0.0013	0.5499	x	x	x	x
Age:BPAQ-Hostility	0.1464	0.0179	0.0001	0.4087	0.2867	0.0003	−0.0037	0.0876^t^	x	x	x	x
Age:PANAS-PA	x	x	x	x	0.2838	0.0004	0.0041	0.1319	x	x	x	x
Age:PANAS-NA	x	x	x	x	0.3489	0.0000	0.0005	0.8460	x	x	x	x
**hCrtsl**												
Age:BSI-Anxiety	0.1349	0.0400	−0.0019	0.5271	0.3386	0.0000	−0.0002	0.8469	x	x	x	x
Age:BSI-Depression	0.0994	0.2465	−0.0023	0.4144	0.3473	0.0000	0.0023	0.0773^t^	x	x	x	x
Age:BSI-Aggression	x	x	x	x	x	x	x	x	x	x	x	x
Age:BPAQ-Physical	0.1257	0.0700	0.0002	0.6495	0.2709	0.0008	0.0000	0.9799	x	x	x	x
Age:BPAQ-Verbal	0.1055	0.1933	0.0002	0.7724	0.1842	0.0458	−0.0004	0.7913	x	x	x	x
Age:BPAQ-Anger	0.1992	0.0004	0.0010	0.0360^*^	0.2982	0.0002	−0.0011	0.3734	x	x	x	x
Age:BPAQ-Hostility	0.1455	0.0216	0.0005	0.3547	0.2384	0.0043	0.0001	0.9689	x	x	x	x
Age:PANAS-PA	x	x	x	x	0.2614	0.0013	0.0011	0.6576	x	x	x	x
Age:PANAS-NA	x	x	x	x	0.3349	0.0000	−0.0017	0.4653	x	x	x	x

t p < 0.1;

* p < 0.05;

** *p < 0.01*.

*T, Testosterone; C, Cortisol; DHEA, Dehydroepiandrosterone; P, Progesterone; E2, Estradiol; sC, salivary C; sT, salivary T; sDHEA, salivary DHEA; sP, salivary P; sE2, salivary E2; hT, hair T; hCrtsl, hair C; hDHEA, hair DHEA; hair P, hP; hair cortisone, hCrtson; BSI, Brief Symptom Inventory; PANAS, Positive and Negative Affect Schedule; BPAQ, Buss-Perry Aggression Questionnaire*.

For hair steroids, significant moderation effects of hCrtsl and hCrtson on the association between age and anger emerged (hCrtsl: *R*^2^ = 0.1992, *p* < 0.001; B = 0.0010, SE = 0.0005, *p* = 0.0360, *R*^2^ change = 0.0190; hCrtson: *R*^2^ = 0.2078, *p* < 0.001; B = 0.0003, SE = 0002, *p* = 0.0331, *R*^2^ change = 0.0191). Furthermore, the association between age and aggression (physical and verbal) was significantly moderated by hP (Physical aggression: *R*^2^ = 0.1502, *p* = 0.0139; B = 0.0044, SE = 0.0020, *p* = 0.0315, *R*^2^ change = 0.0209; Verbal aggression: *R*^2^ = 0.1449, *p* = 0.0197; B = 0.0082, SE = 0.0029, *p* = 0.0044, *R*^2^ change = 0.0372). No significant moderation effects emerged for the association between age and negative emotions for hT and hDHEA as moderators (see Table 3).

## Study 2: men stress 40+

### Participants and procedure

The study was conducted in 2016 and comprised a total of 123 men between the ages of 40 and 75 years. Participants were recruited via web pages and by leaflets distributed in the city of Zurich. To be included in the study, participants had to be generally healthy and German-speaking. After initial inclusion, participants completed the MVEQ (see below) and only participants above a certain threshold score (≥4) for vital exhaustion remained in the study. The participants were characterized as mildly (4–10, *n* = 48), substantially (11–14, *n* = 56), or severely (15–18, *n* = 20) vitally exhausted (Appels et al., 1987; von Känel et al., 2004). The further procedure was the same as described in study 1, including the psychometric testing and a subsequent biological examination, in which saliva and hair samples were obtained under standardized conditions.

Participants had a mean age of 52.7 (*SD* = 8.4) years and a mean body mass index (BMI) of 25.8 (*SD* = 3.9). A total of 11.6% of participants reported being regular smokers. Similar to study 1, a relatively high socioeconomic status was observed, reflected by the mean education level (62.0% reported having a degree from a university or a university of applied sciences) and income level (50.0% earned more than CHF 117,000/year). All participants provided written informed consent, and the study protocol was approved by the Cantonal Ethics Committee of the Canton of Zurich.

### Psychometric measures

This study also employed the BSI-18 and BPAQ as described above.

The short form of the German MVEQ (Kopp et al., 1998; Schnorpfeil et al., 2002) consists of nine items rated on a 3-point Likert scale (2 = yes, 1 = don't know, 0 = no). The VE score ranges from 0 to 18, with participants with a cut-off ≥4 being defined as vitally exhausted. The VE questionnaire showed good internal consistency, with Cronbach's alpha = 0.82.

The Positive and Negative Affect Schedule (PANAS) consists of 20 items rated on a 5-point Likert scale from 1 (very slightly or not at all) to 5 (extremely). Participants were asked how they feel at the moment and had to rate the 20 adjectives (e.g., angry, nervous, strong). The PANAS identifies two subscales, one for positive affect and one for negative affect. Cronbach's alpha is 0.88 for the positive affect subscale and 0.87 for the negative affect subscale.

### Endocrine measures

For this study, the same endocrine measures were analyzed as previously described in study 1, including salivary and hair steroids examined by the same laboratories and reporting similar intra- and inter-assay coefficients for salivary and hair analytes.

### Covariates

To provide the highest possible overlap between analyses, covariates were identified which corresponded to the covariates described in study 1 to the highest possible degree. Similar to Study 1, covariates included were BMI, alcohol and tobacco consumption, medication intake, gum bleeding during the last days, and elevated stress during the last week. Additional covariates were having a cold or infection during the last weeks and general health parameters such as, having a somatic disorder or having age-related symptoms.

### Statistical analysis

The statistical analyses, statistical software and significance level were the same as described for Study 1.

### Results

Sample characteristics and descriptive statistics for salivary and hair analytes are summarized in Table 1. Pearson's correlation analyses showed significant associations between age and different psychometric indicators of negative emotions (see Table 2). The BSI-18 subscale depression was negatively associated with age (*r* = −0.158, *p* = 0.042), while no association emerged for anxiety (*r* = 0.019, *p* = 0.418). With regard to the aggression questionnaire, age was significantly positively associated with the subscales physical and verbal aggression (physical aggression: *r* = 0.171, *p* = 0.030; verbal aggression: *r* = 0.180, *p* = 0.024), but not with anger (*r* = −0.006, *p* = 0.474), or hostility (*r* = −0.022, *p* = 0.407). A positive age-related association emerged for positive affect (*r* = 0.218, *p* = 0.008), while no association was observed for negative affect (*r* = −0.090, *p* = 0.163). Age was negatively associated with sT (*r* = −0.260, *p* = 0.002), sDHEA (*r* = −0.327, *p* < 0.001) and sP (*r* = −0.191, *p* = 0.018) but not with sE2 (*r* = 0.071, *p* = 0.220), and as a trend with sC (*r* = −0.121, *p* = 0.093). When employing partial correlation analyses including the set of covariates introduced for study 1, associations with age did slightly change (anxiety: *r*_*p*_ = −0.110, *p* = 0.131; depression: *r*_*p*_ = −0.117, *p* = 0.094; anger: *r*_*p*_ = 0.131, *p* = 0.090; physical aggression: *r*_*p*_ = 0.160, *p* = 0.051; verbal aggression: *r*_*p*_ = 0.180, *p* = 0.033; positive affect: *r*_*p*_ = 0.149, *p* = 0.064; negative affect: *r*_*p*_ = 0.001, *p* = 0.495; sT: *r*_*p*_ = −0.246, *p* = 0.006; sDHEA: *r*_*p*_ = −0.338, *p* < 0.001; sE2: *r*_*p*_ = 0.008, *p* = 0.468; sP: *r*_*p*_ = −0.193, *p* = 0.024; sC: *r*_*p*_ = −0.125, *p* = 0.101; see Table 2). However, for the hair steroids, a significant association with age emerged only for hT (hT: *r* = −0.169, *p* = 0.050), and a negative trend emerged for hDHEA (hDHEA: *r* = −0.153, *p* = 0.068), while all other hair steroids failed to show age-related associations (hCrtsl: *r* = −0.058, *p* = 0.264; hCrtson: *r* = −0.057, *p* = 267; hP: *r* = −0.102, *p* = 0.161). The pattern changed when including covariates into the correlation analyses, with partial correlation analyses revealing the following age-related associations with hair steroids: hCrtsl: *r*_*p*_ = 0.139, *p* = 0.031; hCrtson: *r*_*p*_ = 0.100, *p* = 0.090; hT: *r*_*p*_ = −0.187, *p* = 0.006; hP: *r*_*p*_ = −0.118, *p* = 0.058; hDHEA: *r*_*p*_ = −0.026, *p* = 0.366.

Moderation analyses for the association between age and parameters of negative emotional state including covariates are summarized for all three studies in Table 3. Significant moderation effects of sC on the association between age and the BSI-18 anxiety emerged (*R*^2^ = 0.4062, *p* < 0.001; B = −0.0152, SE = 0.0067, *p* = 0.0262, *R*^2^ change = 0.0305), while a trend for negative affect (PANAS) was observed (*R*^2^ = 0.4051, *p* < 0.001; B = −0.0242, SE = 0.0139, *p* = 0.0859, *R*^2^ change = 0.0181). For sT, a significant negative moderation effect for the association between age and anxiety (*R*^2^ = 0.4022, *p* < 0.001; B = −0.0027, SE = 0012, *p* = 0.0225, *R*^2^ change = 0.0339) and a trend for depression (*R*^2^ = 0.4070, *p* < 0.001; B = −0.0023, SE = 0.0014, *p* = 0.100, *R*^2^ change = 0.0171) were observed. For sP and sDHEA, no moderation effects were observed, while for sE2, a positive trend for hostility (*R*^2^ = 0.3418, *p* = 0.001; B = 0.1168, SE = 0.0687, *p* = 0.0922, *R*^2^ change = 0.0192) and physical aggression (*R*^2^ = 0.3605, *p* < 0.001; B = 0.0860, SE = 0.0493, *p* = 0.0841, *R*^2^ change = 0.0197) emerged.

For hCrtsl, no significant moderation effects emerged, but a trend was found for the BSI-18 depression scale (hCrtsl: *R*^2^ = 0.3473, *p* < 0.001; B = 0.0023, SE = 0.0013, *p* = 0.077, *R*^2^ change = 0.0196). For hCrtson, two trends for moderation effects resulted for the association of age with verbal aggression (*R*^2^ = 0.1974, *p* = 0.0270; B = −0.0026, SE = 0.0015, *p* = 0.0817, *R*^2^ change = 0.0234) and hostility (*R*^2^ = 0.2867, *p* < 0.001; B = −0.0037, SE = 0.0022, *p* = 0.0876, *R*^2^ change = 0.0200). Furthermore, for hT, a significant positive moderation effect emerged for the association between age and negative affect (PANAS) (*R*^2^ = 0.3719, *p* < 0.001; B = 0.0394, SE = 0.0161, *p* = 0.0168, *R*^2^ change = 0.0462). No moderation effects were observed for hP and hDHEA, although for hDHEA, there was a trend for an association between age and anger (*R*^2^ = 0.3776, *p* < 0.001; B = 0.0119, SE = 0.0060, *p* = 0.0526, *R*^2^ change = 0.0297, see Table 3).

## Study 3: costs and benefits of fatherhood across the lifespan

### Participants and procedure

Study 3 was conducted in the year 2014. Participants for study 3 were recruited within the German-speaking countries of Central Europe (e.g., Austria, Germany, Switzerland) through announcements in daily newspapers, broadcast and online platforms, social networking sites, mailing lists of different family- or research-related organizations, and flyers displayed in public places such as, shopping malls or universities. Inclusion criteria were male sex, a minimum age of 18 years, and having assumed the paternal role for at least one biological or non-biological child in the course of a lifetime. All subjects gave informed consent prior to their study participation. The study protocol was approved by the local Ethics Committee of the Faculty of Arts, University of Zurich, Switzerland.

Data collection was implemented in two steps. First, all subjects completed an (*N* = 2908: original sample). Second, subjects were invited to participate in the follow-up study which included the assessment of salivary endocrine measures. A total of 425 participants subsequently provided saliva samples for the analysis of baseline values of steroid hormones. Of these, 11 subjects had to be excluded due to intake of drugs or anabolic steroids and 30 due to missing information on one of the included covariates, resulting in a final sample of 384 participants. Of those 343 men reported to have fathered only a biological child, while 7 reported to have fathered only a non-biological child and 34 reported to have fathered both. The different forms of fatherhood were not associated with different steroid hormone levels, what is in accordance with previous research (Gray et al., 2017). Participants had a mean age of 43.75 (*SD* = 10.7) years and a mean BMI of 25.3 (*SD* = 3.6). Twelve percentage of the participants reported being regular smokers. In general, a relatively high socioeconomic status was observed, as reflected by the mean education level, with 59% reporting having completed tertiary or higher education. Participants in the sample were relatively healthy, with 91% of participants rating their general health as “outstanding,” “very good,” or “good,” and only 9% considering their health status to be “moderate” or “poor.” Prior published data of this sample can be reviewed here (Ruppen et al., 2016; Waldvogel and Ehlert, 2016, 2017).

### Psychometric measures

Consistent with studies 1 and 2, self-reported psychological distress was measured using the German translation of the BSI-18 and subsequently focusing on anxiety and depression subscales as indicators of negative emotions (Spitzer et al., 2011).

Aggression was measured via the aggressiveness/hostility subscale of the German version of the Brief Symptom Inventory (BSI; Franke et al., 2011). This widely used, reliable, and valid self-report measure assesses symptoms of psychological distress on nine dimensions, including aggressiveness/hostility, during the past 7 days. The subscale aggressiveness/hostility consists of 5 items measuring symptoms of irritability, unbalanced mood or anger, up to strong aggressiveness and hostility on a 5-point Likert scale ranging from 0 (not at all) to 4 (extremely). The five items were aggregated into a sum score ranging from 0 to 20.

### Endocrine measures

Saliva sampling was carried out under standardized procedures during participants' daily routine on 2 consecutive working days directly after awakening. Saliva sampling materials, including two polypropylene tubes and detailed instructions of the sampling procedure were sent to the participants in advance by mail. Participants were asked to refrain from alcoholic and caffeinated beverages, heavy exercise, and sexual activity for 12 h before testing. Moreover, they were instructed not to brush their teeth, chew gum, smoke, eat, or consume non-water beverages for 1 h prior to saliva collection. Mean sampling times were 6:38 a.m. on day 1 and 6:34 a.m. on day 2. The mean awakening time was 6:16 a.m. on both days. On the second sampling day, participants returned both of their samples to our laboratory, where they were immediately stored at −20°C until laboratory analysis at the Biochemical Laboratory of the Psychological Institute of the University of Zurich.

Saliva samples were collected by passive drool using ultra-pure polypropylene sampling devices (SaliCaps: IBL International GmbH, Hamburg, Germany). Steroid hormone concentration for sC, sT, and sE2 was determined in each sample via standard luminescence immunoassays using kits from IBL International GmbH, Hamburg, Germany. Inter- and intra-assay coefficients and analytical sensitivity were similar to in studies 1 and 2. After laboratory analyses, the two values for each participant were averaged to reduce noise in hormone values resulting from pulsatile secretion (Gray et al., 2004), thus providing a more reliable value of basal hormone concentrations.

### Covariates

Similar to study 1, covariates included in the analyses were BMI, alcohol, and tobacco consumption, medication intake, general health status, recent physical activity, recent sexual activity, waking time of the participant, and gum bleeding during the last days. Additional covariate was the time of saliva sampling.

### Statistical analysis

Statistical analyses were conducted using SPSS, version 22.0 (IBM, Armonk, NY) and PROCESS for SPSS, version 2.15 (Hayes and Preacher, 2014). First, correlations between age and negative emotions or steroid hormones, respectively, were calculated using Spearman's rho due to skewed distribution of variables and relatively influential data points representing potential outliers. Spearman correlations are more robust against influential data points by calculating rank order correlations and thereby not including the actual difference between data points. Subsequently, partial correlations with the aforementioned covariates added. Next, similar to studies 1 and 2, moderation analyses were conducted for the association between age and negative emotions, moderated by steroid hormones while potential covariates were included. All statistical tests were two-tailed with statistical significance set at *p* ≤ 0.05.

### Results

Sample characteristics and descriptive statistics of dependent and independent variables are summarized in Table 1. Spearman's correlations showed significant associations between age and negative emotions. Age was negatively associated with aggression (*r* = −0.117, *p* = 0.022) and anxiety (*r* = −0.105, *p* = 0.039), while no association was found between age and depression (*r* = 0.027, *p* = 0.597). Additionally, age was negatively associated with sT (*r* = −0.204, *p* < 0.001) and sC (*r* = −0.104, *p* = 0.042), and positively associated with sE2 (*r* = 0.147, *p* = 0.004).

When considering covariates, associations between age and negative emotions only changed marginally, with aggression and anxiety still being negatively correlated and depression not being associated with age (aggression: *r*_*p*_ = −0.175, *p* = 0.001; anxiety: *r*_*p*_ = −0.113, *p* = 0.030; depression: *r*_*p*_ = −0.011, *p* = 0.832). Associations between age and steroid hormones partly changed after controlling for covariates. While remaining negatively correlated with sT (*r*_*p*_ = −0.176, *p* = 0.001) and positively correlated with sE2 (*r*_*p*_ = 0.151, *p* = 0.004), age was no longer associated with sC (*r*_*p*_ = −0.040, *p* = 0.368).

As presented in Table 3, moderation analyses for the associations between age and negative emotions, including covariates, revealed a trend toward sT moderating the association between age and anxiety (*R*^2^ = 0.117, *p* < 0.001; B = 0.030, SE = 0.015, *p* = 0.051, *R*^2^ change = 0.009). Further, the association between age and depression was found to be moderated by sT (*R*^2^ = 0.146, *p* < 0.001; B = 0.041, SE = 0.018, *p* = 0.021, *R*^2^ change = 0.012; Table 3). No other significant interaction effects for the considered steroids as moderators of the association between age and negative emotions could be revealed in this sample.

## Discussion

In this multi-study report, we intended to replicate findings of increased positive and a decreased negative emotional experience with increasing age (Carstensen et al., 2011; English et al., 2014). Moreover, we aimed to test whether acute levels of steroid hormones measured from saliva, or chronic levels of steroid hormones measured from hair, moderate age-related alterations in emotional experience. Therefore, we investigated emotional experience and steroid secretion in three independent samples of men, which differed with regard to health status, level of exhaustion, and parenthood.

### Age and emotions

Associations were identified between age and emotional experience in adult men. For the 271 SRH men, age was negatively associated with symptoms of anxiety and, as a trend, with symptoms of depression measured with the BSI-18. However, for the 121 VE men, no age-related association emerged for symptoms of anxiety and depression at first glance. For FA men, a negative association emerged between age and symptoms of anxiety (BSI-18) as well as general aggression (measured with the aggression subscale of the full version of the BSI); however, no association with depressive symptoms was observed. Interestingly, large cross-sectional epidemiological studies of Swiss citizens show an age-related increase in subclinical depression, while the percentage of persons with clinically diagnosed depression slightly decreases (Baer et al., 2013). Christensen and colleagues also reported direct age effects on the increase in symptoms of depression and anxiety in 2,622 participants aged between 18 and 79 (Christensen et al., 1999). These results stand in contrast to the finding of an age-related decline in worry frequency in 637 patients with generalized anxiety disorders aged 65 or older (Miloyan and Pachana, 2015). A recent longitudinal study including four large-scale nationwide datasets from different countries shed some light on these contradictory findings by identifying a U-shaped trend for well-being, with a mid-life nadir around the age of 40 (Cheng et al., 2015). Carstensen et al. observed in a longitudinal study a positive curvilinear association between age and positive emotional experience until the age of 64, followed by a flattening of the slope (Carstensen et al., 2011). However, Gana et al. report in another longitudinal study including 899 older subjects (62–95 years) a linear relationship between age and life satisfaction (Gana et al., 2013). Our results for the SRH men are consistent with the assumption of a U-shaped development of positive emotional experience, as the youngest participants in our sample were aged 40. At this age, a nadir of positive emotional experience was shown; thus, a negative linear association of symptoms of anxiety and depression with age further underline such a U-shaped perspective. Furthermore, a negative association between symptoms of anxiety and age was also found in FA men. However, the lack of association for VE men indicates that subgroups suffering from VE might not show an age-related increase in positive emotional experience due to the well-investigated strong association between vital exhaustion and symptoms of anxiety and depression (McGowan et al., 2004; Rafael et al., 2014). The same picture emerged for measures of aggression, with a negative association between aggression measures and age being found in SRH and FA men, although verbal aggression significantly increased with age in SRH men. This might be due to the shift from internalizing negative feelings at younger ages to expressing more negative feelings at older ages. Such a shift could be an important mechanism through which more positive emotions are experienced with age, as studies have reported that the expression of negative emotions is associated with positive relationship outcomes, including elicitation of support, building of new close relationships, and heightening of intimacy in the closest of these relationships (Graham et al., 2008). However, for VE men, measures of aggression were generally positively associated with age, indicating increased anger experience with vital exhaustion, as previously reported (Keltikangas-Järvinen et al., 1996). Nevertheless, as a trend, VE men showed an age-related increase in positive emotions measured with the PANAS, but no decrease in negative affect.

These results are in line with the socioemotional selectivity theory (SST), which describes an increase in the selection of emotional experience with decreasing life expectancy (Carstensen et al., 2003). With increasing age, increased motivation to engage in emotionally meaningful relationships and activities is reported, as well as a preference for attention to and processing of positive over negative information (English and Carstensen, 2014). Evidence in support of this theory stems from a recent experimental study by Mattan et al., who describe a prioritization of self-relevant perspective-taking, despite generally poorer perspective-taking capacity, in older individuals (Mattan et al., 2017). In this experiment employing two visual perspective-taking paradigms, a shift toward a first-person (vs. a third-person) and a self-associated (vs. other-associated) perspective was observed with increasing age. Furthermore, another investigation of the prevalences of three specific emotional profiles (dissatisfied, happy, resilient) in age groups from 64 to 104 years revealed an age-related decrease in the dissatisfied emotional profile (Etxeberria et al., 2017). Moreover, at around the age of 84, a shift from the happy to the resilient emotional profile seems to occur, further supporting the observed positivity effect in the three examined samples of this report, with the age ranges from 40 to 75 years and 25 to 78 years. In addition, the positivity effect representing a favoring of information relevant to emotion-regulatory goals (predominantly goals related to well-being in older individuals) has been replicated in this study in the SRH men and in part FA men (Reed and Carstensen, 2012). The fact that this age-related positivity effect was not observed in VE men might be best explained by the association of vital exhaustion and reduced cognitive resources (Abd-elfattah et al., 2015). Reduced cognitive resources have previously been identified as the main factor reducing positivity (Reed and Carstensen, 2012). Therefore, VE individuals having less cognitive resources potentially do not show the same positivity effect compared to individuals with sufficient cognitive resources.

### Age and steroids

For the sex steroids T, DHEA, and P, a typical age-related decrease was observed in all three samples (Nankin et al., 1981; Feldman et al., 2002; Frost et al., 2013), with also an age-related decrease of E2 in the SRH men but no association with age in the VE men and the exception of an age-related increase in E2 for FA men, which has been reported previously (Berg and Wynne-Edwards, 2001; Gettler et al., 2011; Perini et al., 2012). However, it is important to remark that the sample of FA men was significantly younger and lower sex steroid levels in the FA men are therefore somewhat surprising. The effect of fatherhood might be responsible for this finding. Both sC and hCrtsl were only positively associated with age in SRH men, which is well-described for the general population in the literature (Karlamangla et al., 2013; Nater et al., 2013; Feller et al., 2014; Miller et al., 2016). However, HPA-axis dysregulation is well-established in mood disorders and its heterogeneous expression in hyper- and hypocortisolism might underlie the lack of association between C and age in VE men (Ehlert et al., 2001; Pariante, 2017; Walther et al., 2017c). The lack of association between age and C in FA men is somewhat surprising, although studies report a decline in C levels in expectant fathers (Berg and Wynne-Edwards, 2001), which might have confounded an age-related increase.

### Exploratory analyses and correction for multiple testing

Exploratory analysis was conducted to detect potential associations between age-related alterations in emotional experience and age-related alterations in steroid secretion in men. This is the first study to investigate moderation effects of the steroids T, DHEA, E2, P, and C as well as cortisone on the age-related decrease in negative emotional experience and increase in positive emotional experience. As we used three independent samples, each with slightly different measures of emotional experience, first of all, we identified measures which were employed in all three studies, such as, the BSI subscales for symptoms of anxiety and depression. Furthermore, for the Men's Health 40+ and Men Stress 40+ studies, the aggression questionnaire by Buss and Perry (1992) was used, while the study on costs and benefits of fatherhood across the lifespan used the subscale of the BSI on aggression (Spitzer et al., 2011). Therefore, a large number of tests had to be conducted to test every combination between steroid hormone (up to 10 measures) and measure for emotional experience (up to 9 measures) in each of the three samples. This yielded a total of (10 × 9 × 3–missings) 149 comparisons (see Table 3). The likelihood of finding a significant association by chance increases with the number of tests (type 1 error; rejecting *H*_0_, when *H*_0_ is correct). Alpha error accumulation with 149 comparisons using an alpha level of 0.05 would increase the probability of detecting a false-positive association from the standard 5 to 745%. Therefore, with 149 comparisons, seven to eight significant associations are expected to occur by chance. In fact, a total of nine significant moderation effects emerged. Importantly, adjusting for multiple comparisons reduces the statistical power of studies, a problem that is especially relevant for psychology, behavioral ecology, and animal studies, which usually try to identify small to medium effects due to the multifactorial nature of behavior (Nakagawa, 2004). This means that with correction for multiple testing, the type 2 or beta error rate increases (not rejecting *H*_0_ when *H*_0_ is false). Therefore, correction for multiple testing and blind adherence to particular *p*-values has been criticized under certain conditions such as, exploratory analyses, and researchers have called for a shift toward the inclusion of biological significance, effect sizes, and common sense (Cabin et al., 2017). In our case, when applying the Holm-Bonferroni method (0.5 / (149 – 1 + 1)) (Abdi, 2010), a minimum *p-*value of 0.000335 would be necessary to render an association significant. For the moderation analyses, the smallest *p*-value was 0.0044, indicating no moderation effects of steroid hormones on the association between age and emotional experience at all. However, an analysis of all moderation effects of salivary and hair steroids on age-related alterations in emotional experience yields interesting patterns, which merit further description. Therefore, we followed an exploratory approach, refraining from multiple correction and instead providing an overview of different associations and reporting the 149 comparisons, which may be of interest for further observational or experimental studies (see Table 3).

### Effects of steroids on age-related alterations in emotional experience

In the samples of SRH and VE men, a negative moderation effect of sC on the association between age and symptoms of anxiety emerged. Higher levels of sC were related to a buffered decrease in symptoms of anxiety with age resulting in stable or higher levels of anxiety experience with age. This is in accordance with a multitude of studies reporting higher levels of basal sC levels in patients with generalized anxiety disorder (Mantella et al., 2008) or other forms of anxiety disorders (Vreeburg et al., 2010). The anxiogenic effects of C administration are also well established (Ardayfio and Kim, 2006; Murray et al., 2008). Interestingly, this was not observed for fathers, with a diminished effect of C being found in FA men, or a floor effect due to lower levels of C due to fatherhood. However, the sampling method differed between the studies: For the SRH and VE men, saliva samples were obtained under standardized conditions during a biological examination performed by well-trained personal at 8:00 am. By contrast, FA men collected their saliva at home, mostly for the first time, which might be the reason for the differences between these groups. In addition, for hCrtsl and hCrtson (a metabolite of C), no moderation effects were detected for the association between age and symptoms of anxiety. However, this has been reported by previous studies investigating associations between patients with anxiety disorders and hCrstl (Steudte et al., 2011; Steudte-Schmiedgen et al., 2017).

sT was a significant moderator of the association between age and symptoms of depression in FA men, indicating an anti-depressant effect of T in the course of aging in these men. A multitude of studies have reported anti-depressant effects of T in mice (McHenry et al., 2014; Carrier et al., 2015), in different subgroups of men (Johnson et al., 2013; Walther et al., 2017c), and especially in older men (Snyder et al., 2016; Walther et al., 2016). However, for healthy men, a lack of association between depressive symptoms and T is often reported (Johnson et al., 2013), and a recently published review stated that evidence for the effectiveness of testosterone treatment in men with low T is lacking (Huo et al., 2016), thus potentially explaining the lack of moderation effect of T and symptoms of depression in SRH men. Furthermore, the lack of association in VE men, who primarily have low levels of vitality, might also be explained by the finding of Snyder et al. (2016) that T treatment in older men had no effect on vitality or physical performance. Nevertheless, salivary T moderated the association between age and symptoms of anxiety in the VE men, and hT moderated the association between age and negative affect. Previous research has demonstrated that T exerts anxiolytic effects (Mahmoud et al., 2016; Wainwright et al., 2016), and that these effects might be mediated in part by the aromatization to E2 (Carrier et al., 2015). However, no moderation effects of sE2 on age-related alterations in emotional experience were observed in any of the samples. Recent research showed that endogenous T mediated the association between cerebellar gray matter and measures of neuroticism, and that higher T levels were associated with lower scores for neurotic personality and larger cerebellar gray matter volumes (Schutter et al., 2017). This provides a cerebellum-oriented framework for the susceptibility to experience negative emotions and mood, moderated by T, thus further strengthening our findings regarding the moderation effect of T on age-related emotional experience.

No moderation effects emerged for DHEA, either for saliva sampling or hair sampling, which contributes further to the list of positive, negative, and null findings related to DHEA and mood or emotional experience in general (Kudielka et al., 1998; Wolf et al., 1998; Izawa et al., 2008; Heald et al., 2017).

With regard to aggression, sP just failed to show significant moderation effects on the age-related decrease in aggression in SRH men, although trends emerged. However, hP seems to exert significant moderation effects on the association between age and physical and verbal aggression in SRH men, but not in the other samples of men. A similar picture can be observed for hCrtsl and hCrtson on the measures for anger. P is known to have stress-reducing effects and is associated with better mood ratings after psychosocial stress (Childs et al., 2010), as well as reduced cue-induced craving (Fox et al., 2013). However, the association between P and aggression seems to be negative, and in women, high levels of luteal P were associated with low levels of premenstrual aggressive behavior (Ziomkiewicz et al., 2012). For SRH men, physical aggression decreases with age while verbal aggression increases, and hP shows a positive moderation effect for both age-related alterations. Thus, we assume an intensifying effect of P on age-related alterations in aggression expression. Moreover, as verbal aggression has been suggested as a stress-relieving mechanism, higher P levels seem to contribute to a better (more positive) emotional experience with increasing age in healthy men.

In SRH men, hCrtsl and hCrtson were also significant moderators of the association between age and anger. As anger decreases with age in SRH men, higher levels of hCrtsl and hCrtson seem to intensify this association. Studies have linked aggression to increased levels of C in children, with findings of higher levels of C in boys after playing a violent video game (Gentile et al., 2017), increased levels of C in boys with an aggressive form of conduct disorder as well as chronic reactive aggression (Bokhoven et al., 2005), and in boys and girls who had been exposed to violence (Peckins et al., 2012). However, as C increases continuously with age, and has been shown to exert a regulatory function on emotion processing (Lam et al., 2009), it might have an intensifying effect on the age-related decrease in anger perception in healthy men.

### Interaction of steroid hormones affecting emotional experience

Steroid hormones act as a complex network of reciprocally inhibiting and facilitating agents. Therefore, researchers argue that inclusion and analysis of multiple steroid hormones in studies is of additional value and that combined factors such as, hormone ratios provide additional information about the interaction and balance between certain hormones (Sollberger and Ehlert, 2016). If not investigating hormone interactions but the effects of single hormones such as, cortisol in gender-mixed samples, there is also the recommendation to adjust for sex hormones because of their influencing effect on cortisol secretion (Juster et al., 2016). Furthermore, recent research presents evidence for a dual-hormone hypothesis on testosterone and cortisol. This hypothesis suggests that higher levels of testosterone increase dominance, aggressive, and mating behavior only, if cortisol is low and if, in turn, cortisol is present at high concentrations, higher levels of testosterone decrease the afore mentioned behaviors (Terburg et al., 2009; Mehta and Josephs, 2010; Van Anders, 2013). Therefore, in the presented study higher order moderations were calculated to check for potential interaction effects of steroid hormones on the association between age and emotional experience, but no significant associations emerged (data not shown).

### Similarities and differences between the samples

On average, the three samples showed high levels of education and income. Therefore, the results from the three independent studies can primarily be generalized to men with a medium to high socioeconomic status. The sample from the Men's Health Study is, on average, 5 years older than the sample from the Men Stress Study, even though the two studies recruited men from the same age range. SRH and FA men showed similar levels of experienced anxiety and depression, while VE men reported higher levels of depressive and anxiety symptoms on average. However, SRH and VE men showed similar levels of experienced aggression. Based on the younger average age of the Men Stress Study sample, one might expect higher levels of sex steroids in the Men Stress Study due to age-related sex steroid decline. However, this was not observed for the salivary sex steroids, possibly due to the overall health status of the men from the Men Stress Study, who had elevated levels of vital exhaustion. Good general health has been shown to reduce or even prevent age-related sex steroid decline (Feldman et al., 2002; Sartorius et al., 2012; Walther et al., 2016), while mortality is associated with a decline in sex steroids (Hsu et al., 2016). Furthermore, a male-specific pattern for mood disorders has been suggested, with overall decreased androgen levels (Walther et al., 2017c), while for FA men, lower levels of androgens were expected due to fatherhood, as observed in the third sample (Perini et al., 2012; Waldvogel and Ehlert, 2016). However, for C, a conflicting picture emerged with regard to saliva and hair samples. However, as mentioned above the comparability of FA men with the SRH and VE men is limited due to the different age range and different sampling methods for the salivary analytes. Though FA men are in average younger than the SRH and VE men they show lower levels of sT. This might be explained by their role as a father, which is associated with reduced T levels and outweigh the effect of age. SRH and FA men showed similar levels of sC, while VE men showed relatively low levels of sC in comparison. This has previously been reported in patients with clinical burnout, for whom exhaustion plays a major role (Lennartsson et al., 2015). Furthermore, mean hCrtsl and hCrtson levels were higher in VE men than in SRH men, indicating that vital exhaustion is associated with elevated hair C and cortisone. This is in line with previous studies demonstrating higher levels of C in related conditions such as, burnout or depression (Ehlert et al., 2001; Menke et al., 2014; Pariante, 2017).

### Similarities and differences between men and women

The question whether similar effects would be observed in female samples based on the presented results is hard to answer because of three major differences between male and female endocrinology. First, women undergo endocrine changes during menopause in a much more condensed time period (average age of starting menopause is 51 years with an average duration of 4–10 years) and in a much more dramatic way compared to men (abrupt decline of estrogens and progesterones vs. continuous decline of androgens). Therefore, women over 40 years can't be regarded as a homogeneous group with regard to their circulating sex hormone levels and subgroups of pre-, (peri-,) and post-menopausal women need to be examined separately in order to dissect the effects of steroid hormones for each subgroup individually as shown previously (Drobnjak et al., 2014). Second, circulating levels of sex steroids are significantly different between sexes showing for example up to 10 times higher androgen levels in males raising the question of physiological relevant hormone levels for certain steroids to act as neuroactive steroids. And third, during the fetal development the male and female brain are differentially programmed because of the different amounts of sex steroids secreted by the fetus and circulating in the womb (Seckl, 1998; Schore, 2017). Therefore, different levels of steroid hormones might have similar effects in men and women due to differential sensitivity to certain hormones represented by differences in steroid receptor distribution or function. However, since the menopausal transition is characterized by a decline of estrogen and progesterone it could be assumed that lower levels of estrogen and progesterone contribute to the observed changes in emotional experience. However, studies investigating estrogen and progesterone replacement in post-menopausal women contrast this hypothesis by reporting beneficial effects on mood with hormone replacement therapies (Fitzpatrick et al., 2000; Gleason et al., 2015). Therefore, studies investigating the moderating effects of steroid hormones on the age-related alterations in emotional experience in women are needed.

## Limitations

Several limitations need to be taken into account when interpreting these results. The three studies employed cross-sectional designs and causal inferences cannot be drawn. Whether or not certain steroid hormones intensify age-related changes in emotional experience cannot be determined from the present findings. Only associations were identified, although these associations also need to be interpreted with caution, as we conducted a multitude of comparisons in an exclusively exploratory manner, without correction for multiple testing. Identified moderation effects might serve as initial starting points to further investigate the potential effects of steroids on age-related alterations in emotional experience using experimental designs. Furthermore, saliva sampling differed in one study, potentially increasing measurement error. Salivary analytes were obtained via a single measurement time point on a single day. However, salivary steroid hormones tend to vary significantly between days. Repeated measures on consecutive days would increase the robustness of the results and reduce between-day variance in steroid secretion. Furthermore, on average, psychometric measures were obtained 1 to 2 weeks before sampling of the hormone parameters. Experimental research designs measuring emotional experience in different age groups, manipulating hormone levels in a controlled manner, are needed to further test the revealed associations and identify causality.

## Conclusion

Age-related alterations in emotional experience were identified in three independent samples of men. In support of the SST and the age-related positivity effect, a general shift toward more positive emotions was identified for SRH and FA men with increasing age, although this was not found for VE men, in whom detrimental effects of vital exhaustion on emotional experience in aging men emerged. Several potential moderation effects of steroid hormones were described, which merit further investigation. Since moderation effects faded after controlling for multiple comparisons, single significant results on emotional experience need to be replicated in experimental studies manipulating hormone levels systematically in different age groups before drawing final conclusions. Hormonal supplementation or the use of antagonists has been shown to support healthy aging in men and women (Lunenfeld et al., 2013; Walther and Ehlert, 2015; Hamoda et al., 2016), and might further support a successful aging process in men by intensifying the positivity shift with age. However, to date there is not sufficient empirical evidence for hormonal supplementation or antagonists facilitating the age-related positivity shift. Furthermore, the presented results are generalizable to adult males up to 75 years of age only while with respect to women, which differ substantially in basal steroid levels or secretion patterns from men, no conclusions can be drawn. Still, as emotional experience directly affects morbidity and mortality (Carney et al., 2002; Kiecolt-glaser et al., 2002; Carstensen et al., 2011; Steptoe and Wardle, 2011), it should be a public health goal to increase positive emotional experience. Therefore, more research is needed to elucidate the role of steroid hormones on the age-related shift toward more positive emotional experience.

## Author contributions

AW, PW, EN, JR, and UE contributed equally to the design of the study and the data collection, analysis, and interpretation of the data. AW wrote the first draft of the manuscript. PW wrote the specific sections of study 3 and was leading for the representation of data in the tables. EN and JR edited subsequent versions of the manuscript. UE reviewed the manuscript during different stages of the process for intellectual content and edited the manuscript to its final version. All authors approved the final version of the manuscript.

### Conflict of interest statement

The authors declare that the research was conducted in the absence of any commercial or financial relationships that could be construed as a potential conflict of interest.

## Abbreviations

SRH: Self-reporting healthy
VE: Vital exhaustion
FA: fathers
T: Testosterone
C: Cortisol
DHEA: Dehydroepiandrosterone
P: Progesterone
E2: Estradiol
sC: salivary C
sT: salivary T
sDHEA: salivary DHEA
sP: salivary P
sE2: salivary E2
hT: hair T
hCrtsl: hair C
hDHEA: hair DHEA
hP: hair P
hCrtson: hair cortisone
HPA: hypothalamic-pituitary-adrenal
HPG: hypothalamic-pituitary-gonadal
BSI: Brief Symptom Inventory
PANAS: Positive and Negative Affect Schedule
BPAQ: Buss-Perry Aggression Questionnaire
MVEQ: Maastricht Vital Exhaustion Questionnaire.
